# Functional Equations for the Enhancement Factors for CO_2_-Free Moist Air

**DOI:** 10.6028/jres.080A.007

**Published:** 1976-02-01

**Authors:** Lewis Greenspan

**Affiliations:** Institute for Basic Standards, National Bureau of Standards, Washington, D.C. 20234

**Keywords:** Enhancement factor, moist air, saturated air

## Abstract

Equations are presented which explicitly express the enhancement of water vapor in CO_2_-free air from 0.1 to 2 MPa. The equations are approximations to the formulation of Hyland and provide the means of obtaining enhancement with very modest computational facilities. The agreement with Hyland’s enhancement values is well within his estimated uncertainty.

## 1. Introduction

The effective saturation pressure of water vapor in equilibrium with a plane surface of liquid or solid water in the presence of an admixed gas above its critical temperature differs from the saturation vapor pressure of the pure phase. It is expressed by

$$f=\frac{x_{w}P}{e_{s}}$$ (1)

where

*f*: = the enhancement factor
*x_w_*: = the mole fraction of water vapor in the saturated mixture.
*P*: = total pressure above the surface of the condensed phase (liquid or solid)
*e_s_*: = the pure phase saturation vapor pressure of water.

In the field of humidity it is important to know both *e_s_* and *f* in order to determine the quantity of saturated water vapor in a gas. As an example, the mixing ratio of a gas saturated with water vapor is given by

$$r=\frac{\epsilon fe_{s}}{P-fe_{s}}$$ (2)

where

*r*: = the mixing ratio in mass of water vapor per unit mass of associated dry gas.
*ϵ*: = the ratio of the molecular weight of water to the molecular weight of the associated dry gas.

Whereas *e_s_* is and has been known over a fairly wide range of temperature to a high degree of accuracy, accurate values of *f* have not been available for any gas until Hyland [1]^1^ recently published values of *f* for water vapor in CO_2_-free air over a wide range of temperatures and pressures. These values are based on experiments from which the approximate theoretical form for the second interaction (cross) virial coefficient for air-water vapor mixtures was obtained. Use of this coefficient in a thermodynamically based equation permits determination of the enhancement factor. This equation, though implicit in *f*, can be used in an iterative mode to calculate *f* over fairly wide ranges of temperature and pressure. The equation is complex and implicit in *f* and therefore requires high speed computer capability for the determination of *f* at any given temperature and pressure. An alternate and simpler method of obtaining values of *f* for specific conditions would be by interpolation in tables first obtained by computer from Hyland’s equation. Since a two way (temperature and pressure) interpolation of a function nonlinear in two parameters would be required, such interpolation would, at best, be awkward unless the table were close spaced.

By generalizing an equation, originally due to Goff and Gratch [2] and fitting it to values obtained from Hyland’s relation, we have obtained simplified explicit equations for *f* which can be easily programed for a computer or can be calculated with the aid of a pocket calculator.

## 2. Method

Goff and Gratch [2] used the following form of equation for *f*

$$f=\exp\;\left[\alpha \left(1-\frac{e_{s}}{P}\right)+\beta \left(\frac{P}{e_{s}}-1\right)\right]$$ (3)

where *α* and *β* were obtained from theoretical equations and presented in a table of *α* and *β* for water and ice as a function of temperature. The implication was that *α* and *β* were independent of pressure, which is not precisely true, but they are fairly insensitive over modest pressure ranges.

We obtained values of *α* and *β* based on the new data given by Hyland. We calculated these values of *α* and *β* for water at temperatures from −50 to 100 °C and pressures from 0.101325 MPa (1 atm) to 2.0265 MPa (20 atm) and for ice from −100 to 0 °C over the same pressure range.

*α* and *β* were obtained by a least square fit of Hyland’s values to equation

$$\frac{P}{P-e}\text{Ln}\;f=\alpha +\beta \frac{P}{e}$$ (4)

at 20 equally spaced values of pressure from 0.101325 MPa to 2.0265 MPa for every 2 deg C.

We then fitted these values of *α* and *β* as a function of temperature, a minimum of 26 points being used for any *α* or *β* fit. The result was

$$\alpha =\sum_{i=1}^{4}A_{i}t^{\left(i-1\right)}$$ (5)

and

$$\beta =\exp\sum_{i=1}^{4}B_{i}t^{\left(i-1\right)}$$ (6)

where *t* is in degrees Celsius IPTS 68.

Table 1 gives the values of *A_i_* and *B_i_* for three conditions at 0.1 to 2 MPa.

The agreement between these equations and values obtained with the Hyland formulation is shown in table 2.

It is of interest to compare deviations between these formulations and Hyland’s formulation with his estimate of the total uncertainty. This comparison is shown in table 3 for the range in which Hyland has given an estimated uncertainty. The deviations nowhere exceed Hyland’s estimated uncertainties and in general are more than an order of magnitude less than the uncertainties.

It will be noted that these formulations have been compared with Hyland’s formulation in regions where Hyland has declined to assign uncertainties (below −50 °C and above 90 °C as well as in regions where he has published no values (below −80 °C, above 90 °C and for water below 0 °C). Despite the questionable validity of his formulation in these areas, namely the region of supercooled water and the region of ice below −50 °C, there is no formulation for *f* in these regions that is more valid at this time. It is our suggestion therefore that the Hyland formulation be used for all water-air *f* factors up to the boiling point. It is for this reason that we have proposed our simplified equations for this wide region.

As can be seen from table 2, the agreement between these formulations is exceedingly good above 0 °C and quite good for water down to −50 °C. Because there does not appear to be any need for *f* factors for supercooled water below −50 °C, we have terminated our equation at that point.

There is a need for *f* factors for ice below −50 °C and we have therefore extended our formulation to −100 °C. As can be seen from table 2, the agreement with Hyland is considerably poorer for ice than for water. The agreement can be improved by using two sets of coefficients, one for 0 to −50 °C and the other for −50 to −100 °C, in the same form of the equation. The coefficients, are given in table 4 and the deviations are given in table 5. For the alternate equations, table 3 is not applicable below 0 °C and table 6 is the applicable section of that table.

## 3. Discussion

We have attempted to present equations that will be useful to a wide range of workers in the field of humidity measurement. We have used a historical form of the equation which appears to give reasonably adequate results up to 2. MPa.

It should be pointed out that the deviations between the values obtained from these equations and the values obtained from the Hyland formulation are not random. These equations therefore are not an alternative expression of the Hyland formulation, but merely a convenience for obtaining approximate values of Hyland’s *f* factor.

Of course, there are those who have need for Hyland’s results above 2 MPa, and those people we have not helped. It is regrettable that we required three equations to cover the desired range (some may feel that the four equations are necessary) and that each of the equations has eight coefficients. We do not suggest that the equations presented here are the optimum equations available, but only that they appear adequate for most purposes and appropriate for adoption where uniform simple computations are desirable. It must also be pointed out that these formulations are based on the 1968 IPTS temperature scale, the Wexler [3] formulation for the vapor pressure of water and the Goff [4] formulation for the vapor pressure of ice, which is completely consistent with the work of Hyland. Were new vapor pressure equations to be used which give values that differ markedly from the referenced equations, these formulations would no longer be acceptable. It is important to be aware that under the same circumstances, the values of *f* calculated with Hyland’s formulation would differ from his published values.

## Figures and Tables

**Table 1:** Coefficients for *f*

	Water −50 to 0 °C	Water 0 to 100 °C	Ice −100 to 0 °C
A1	3.62183 × 10^−4^	3.53624 × 10^−4^	3.64449 × 10^−4^
A2	2.60553 ×10^−5^	2.93228 × 10^−5^	2.93631 × 10^−5^
A3	3.86501 × 10^−7^	2.61474 × 10^−7^	4.88635 × 10^−7^
A4	3.82449 × 10^−9^	8.57538 × 10^−9^	4.36543 × 10^−9^
B1	−10.7604	−10.7588	−10.7271
B2	6.39725 × 10^−2^	6.32529 × 10^−2^	7.61989 × 10^−2^
B3	−2.63416 × 10^−4^	−2.53591 × 10^−4^	−1.74771 × 10^−4^
B4	1.67254 × 10^−6^	6.33784 × 10^−7^	2.46721 × 10^−6^

**Table 2:** Summary of deviations of *f* from Hyland formulation

Temp ° C	Water Deviation, %		Ice Deviations, %	
	Sigma	Max	Sigma	Max
100	0.003	0.004		
90	.002	−0.004		
80	.003	−0.006		
70	.001	−0.003		
60	.002	0.003		
50	.004	0.005		
40	.003	0.004		
30	.002	−0.005		
20	.005	−0.013		
10	.006	−0.014		
0	.008	0.011		
−0	.007	−0.009	0.059	0.008
−10	.006	−0.014	.020	−0.042
−20	.008	−0.015	.038	−0.076
−30	.011	−0.020	.020	−0.046
−40	.014	−0.029	.024	0.033
−50	.019	−0.035	.052	0.068
−60			.051	0.069
−70			.032	−0.060
−80			.083	−0.196
−90			.109	−0.259
−100			.163	0.222

**Table 3:** Individual deviations from Hyland formulation

T °C								
*P*	90	70	50	30	10	−10	−30	−50
MPa	Deviations, percent							
0.1	0.001 (0.001)	−0.001 (0.01)	−0.003 (0.01)	−0.004 (0.01)	−0.006 (0.03)	−0.012 (0.04)	−0.021 (0.05)	−0.029 (0.06)
.2	0.000 (0.01)	−0.001 (0.02)	−0.002 (0.03)	−0.003 (0.03)	−0.005 (0.06)	−0.010 (0.07)	−0.016 (0.10)	−0.014 (0.13)
.3	−0.001 (0.02)	−0.001 (0.03)	−0.001 (0.05)	−0.002 (0.04)	−0.003 (0.09)	−0.009 (0.11)	−0.012 (0.14)	−0.001 (0.19)
.4	−0.001 (0.03)	0.000 (0.05)	0.000 (0.06)	−0.001 (0.05)	−0.002 (0.11)	−0.008 (0.15)	−0.009 (0.19)	0.011 (0.25)
.5	−0.001 (0.04)	0.000 (0.06)	0.001 (0.08)	0.000 (0.07)	−0.001 (0.14)	−0.007 (0.18)	−0.006 (0.24)	0.022 (0.32)
1.	−0.001 (0.09)	0.000 (0.13)	0.004 (0.17)	0.002 (0.13)	0.001 (0.29)	−0.009 (0.37)	−0.003 (0.48)	0.059 (0.64)
2.	−0.004 (0.20)	−0.003 (0.26)	0.003 (0.35)	−0.005 (0.27)	−0.014 (0.58)	−0.042 (0.75)	−0.046 (0.98)	0.047 (1.30)

Figures in () are Hyland’s estimated uncertainties in percent for the same conditions as the deviation above.

**Table 4:** Coefficients for Ice Eauation for *f* (alternative form)

	−100 to−50 °C	−50 to 0°C
A1	9.88896 × 10^−4^	3.61345 × 10^−4^
A2	5.74491 × 10^−5^	2.9465 × 10^−5^
A3	8.90422 × 10^−7^	5.21676 × 10^−7^
A4	6.20355 × 10^−9^	5.01622 × 10^−9^
B1	−10.4148	−10.7401
B2	9.11735 × 10^−2^	7.36812 × 10^−2^
B3	5.14117 × 10^−5^	−2.68806 × 10^−4^
B4	3.55087 × 10^−6^	1.53964 × 10^−6^

**Table 5:** Summary of deviations of *f* from Hyland formulation (alternative form)

Temp	Ice Deviation, %	

°C	Sigma	Max
0	0.005	−0.009
−10	.006	−.013
−20	.008	−.014
−30	.010	−.019
−40	.013	−.027
−50	.018	−.033
−60	.024	−.052
−70	.032	−.059
−80	.043	−.080
−90	.060	−.126
−100	.092	−.148

**Table 6:** Individual deviations from Hyland formulation (alternative form)

*P*	T °C		

MPa	−10	−30	−50

	Deviations, %		
0.1	−0.011 (0.04)	−0.019 (0.05)	−0.033 (0.06)
.2	−0.008 (0.07)	−0.013 (0.10)	−0.022 (0.13)
.3	−0.005 (0.11)	−0.008 (0.14)	−0.013 (0.19)
.4	−0.002 (0.15)	−0.003 (0.19)	−0.004 (0.25)
.5	0.000 (0.18)	0.001 (0.24)	0.003 (0.32)
1.	0.006 (0.37)	0.012 (0.48)	0.021 (0.64)
2.	−0.013 (0.75)	−0.018 (0.98)	−0.028 (1.30)

Figures in () are Hyland’s estimated uncertainties in percent for the same conditions as the deviation above.

## Footnotes

1 Figures in brackets indicate the literature references at the end of this paper.
